# Retraction: Evaluating modern intrusion detection methods in the face of Gen V multi-vector attacks with fuzzy AHP-TOPSIS

**DOI:** 10.1371/journal.pone.0352216

**Published:** 2026-06-23

**Authors:** The PLOS One Editors

After this article [1] was published, the author notified the *PLOS One* Editors about multiple errors in the published tables and figures.

Upon editorial assessment, similarities were identified between this article [1] and a previously published article by another author group [2] (cited as reference 19 in [1]). Both studies report the use of a fuzzy AHP-TOPSIS approach to evaluate intrusion detection methods/systems. Substantial overlap was noted between Tables 4, 5, 6, 7, 9, 10, 11, 12, 13, 14, and 15 in this article [1], and Tables 5, 6, 7, 8, 10, 11, 12, 13, 14, 15, and 16 in [2], though some values differed.

During editorial follow-up the author stated that the overlap was unintentional and likely arose owing to their collaboration with the group that authored the previous article [2].

Replacement data for the tables were provided, but upon consultation with a member of the Editorial Board, the *PLOS One* Editors determined that the provided data did not resolve the concerns about the extent of the overlap.

In addition, the *PLOS One* Editors identified concerns about the article’s peer review. We regret that the issues were not addressed prior to the article’s publication.

In light of concerns about overlap with previously published work and about the validity and provenance of the reported results, the *PLOS One* Editors retract this article.

The author did not respond to the final editorial decision.

Tables 4, 5, 6, 7, 9, 10, 11, 12, 13, 14, and 15 report material previously published in [2] under a Creative Commons Attribution (CC BY 4.0) license (https://creativecommons.org/licenses/by/4.0/). The reused content was modified in places. These tables are subject to the license that applies to the original article.
